# Revitalization of the Endophytic Fungus *Acremonium* sp. MEP2000 and Its Impact on the Growth and Accumulation of Bioactive Compounds in *Inonotus obliquus*

**DOI:** 10.4014/jmb.2410.10037

**Published:** 2025-02-13

**Authors:** Hui Wu, Qiao Liu, Limei Zeng, Junzhi Peng, Xinrui Yao, Tao Zhou, Zhiwu Tan, Weifan Wu, Yong Sun, Jihong Jiang, Xiaoying Cao

**Affiliations:** Key Laboratory of Biotechnology for Medicinal Plants of Jiangsu Province, School of Life Sciences, Jiangsu Normal University, Xuzhou 221116, P.P. China

**Keywords:** *I. obliquus*, endophytic fungi, *Acremonium*, growth promotion, secondary metabolites

## Abstract

*Inonotus obliquus* is a large, medicinal and edible fungus known for its extremely slow growth. It has garnered significant attention due to its high socioeconomic and pharmaceutical value. Our research group previously isolated an endophytic fungus, *Acremonium* sp. MEP2000, from *I. obliquus*, and a fermentation broth of MEP2000 enhanced both the growth and bioactive compound accumulation in *I. obliquus*. However, the activity of MEP2000 gradually declined after successive subcultures on PDA medium. We therefore revitalized MEP2000 by supplementing the PDA medium with birch bark powder (H medium), *I. obliquus* fruiting body powder (Z medium), and a combination of both (H&Z medium). MEP2000 was cultured on the PDA medium as the unrejuvenated control (PDA medium). The rejuvenated MEP2000 broth exerted significant effects on the secondary metabolite production, colony morphology, and growth rate of *I. obliquus*, with the H&Z medium inducing the most uniform mycelial morphology and fastest growth. Similarly, liquid shake-flask fermentation experiments showed that the H&Z inducer had the strongest growth-promoting effect. *I. obliquus* intracellular polysaccharide content was also highest in the H&Z treatment, with polysaccharide synthesis-related gene expression following a similar trend. GC-MS analysis confirmed that the H&Z medium had the highest concentration of triterpenoid bioactive compounds, such as inotodiol. These findings suggest that the MEP2000 broth, revitalized using the H&Z medium, effectively promotes *I. obliquus* mycelial growth and bioactive compound production, providing a valuable reference for large-scale production of active endophytic fungi.

## Introduction

*Inonotus obliquus*, a medicinal and edible fungus belonging to the phylum Basidiomycota, class Agaricomycetes, order Hymenochaetales, and family Hymenochaetaceae, holds significant therapeutic value. Commonly referred to as chaga, birch mushroom, or Siberian Ganoderma due to its growth on birch trees, *I. obliquus* is widely distributed across Siberia, the Far East of Russia, northern Europe (including Poland and the Netherlands), Hokkaido in Japan, northern North America, and several provinces and autonomous regions of China, such as Heilongjiang, Jilin, and Inner Mongolia . This fungus is rich in bioactive compounds, primarily polysaccharides, triterpenoids, flavonoids, and polyphenols. Numerous pharmacological and clinical studies have shown that *I. obliquus* exhibits a range of health benefits, including anti-tumor [7], antiviral [8], antioxidant [9], anti-inflammatory [10], blood glucose-regulating [11, 12], lipid-lowering [13], and immune-modulating effects [14]. With the expansion of the medicinal range of *I. obliquus*, the wild resources of *I. obliquus* have been gradually reduced, and it is now difficult to meet market demand. At the same time, artificially cultured *I. obliquus* grows slowly, and the content of active ingredients is lower. Studying and utilizing the endophyte resources in *I. obliquus* has therefore become an effective new way to promote the growth of *I. obliquus* and the accumulation of effective components.

Endophytes refer to a group of microorganisms that live in healthy tissues or organs, as well as in intercellular spaces, during some or all stages of their life history without causing host diseases. They play an important role in promoting biological growth and development, as well as stress resistance. Some endophytes can also produce active secondary metabolites, including alkaloids, flavonoids, saponins, peptides, phenolic acids, terpenes, and other active compounds, which are considered promising sources of new compounds. For example, endophytic fungi in *Taxus chinensis* can produce taxol and the yield of taxol produced by the *Metarhizium anisopliae*, H-27 was 846.1 μg/l in reformative potato dextrose liquid medium [20]. In addition, endophytic fungi *Fusarium solani* S-019 isolated from *Camptotheca acuminata* displayed impressive cytotoxic activity on cancer cells and was found to produce camptothecin by using TLC, HPLC and EI-MS analysis methods [21]. Moreover, from 2017 to 2019, a total of 449 new secondary metabolites were isolated from plant endophytic fungi , which have become a research hotspot as a result. However, strain degeneration is a particularly serious problem in industrial microbial production.

Strain degradation is mainly manifested as slow mycelial growth, weakened resistance, decreased yield of biomass and secondary metabolites, all of which significantly impact the application of endophytes . Our research group previously isolated *Acremonium* sp. MEP2000, an endophytic fungus from *I. obliquus*, which was found to promote both the growth and bioactive compound production in its host. However, the activity of MEP2000 declined with continuous subculturing on PDA medium. In the present study, we therefore sought to restore the growth-promoting and bioactive compound-synthesizing capacity of *Acremonium* sp. MEP2000 by modifying the culture medium composition. Our findings offer valuable insights for overcoming strain degradation and improving yields in the large-scale production of endophytic fungi.

## Materials and Methods

### Strains and Culture Media

The *I. obliquus* strain MDJCBS88, provided by the National Key Laboratory of Biotechnology for Medicinal and Edible Plants at Jiangsu Normal University, was originally isolated from a wild sclerotium collected in the Changbai Mountains of northeastern China. Its identity was confirmed through ITS sequence analysis (GenBank Accession No. DQ103883). The *Acremonium* sp. strain MEP2000, isolated from a wild sclerotium of *I. obliquus* and preserved by the same laboratory, has its ITS sequence registered under GenBank Accession No. KJ777534 .

The PDA medium was prepared by boiling 200 g of fresh, peeled potatoes in 1,000 ml of distilled water, followed by the addition of 20 g of D-glucose and 20 g of agar. The natural pH of the medium was maintained, and sterilization was carried out at 115°C for 30 min.

The PDB medium was prepared by boiling 200 g of fresh, peeled potatoes in 1,000 ml of distilled water, followed by the addition of 20 g of D-glucose. The natural pH of the medium was maintained, and sterilization was carried out at 115°C for 30 min.

The rejuvenation media were formulated by modifying the PDA medium. Specifically, 6 g/l of birch bark powder was added to create H medium, while 6 g/l of *I. obliquus* fruiting body powder was added for Z medium, and a combination of both (6 g/l of each) was used to prepare the H&Z medium.

### Revitalization of MEP2000 and Preparation of Fermentation Broth Inducers

MEP2000, a fungus previously isolated by us for its ability to promote *I. obliquus* growth, gradually lost its growth-promoting activity after successive rounds of artificial cultivation. To address this, MEP2000 was cultured on three types of rejuvenation media, with pure PDA medium serving as the unrejuvenated control. Each group consisted of five replicates, and after 18 days of cultivation, the strain was subcultured for a total of five passages. Observations were made every 72 h, with colony diameter measured using the cross-sectional method to calculate mycelial growth rate and assess mycelial vigor .

After five rounds of subculturing on the four different media, mycelial plugs were extracted from MEP2000 colonies using a 0.5 cm diameter agar punch. The plugs were taken from four diagonally opposite edges of each colony and then inoculated into 50 ml of PDA liquid medium. These cultures were incubated at 28°C with shaking at 160 rpm for 10 days. After incubation, the cultures were vacuum filtered to separate the mycelia, and the resulting filtrate was collected as the fermentation broth. The fermentation broths were sterilized by wet heat at 115°C for 30 min, creating four types of MEP2000 fermentation broth inducers: H inducer, Z inducer, H&Z inducer, and PDA inducer.

### Preparation of *I. obliquus* Seed Culture

*I. obliquus* stored on slants was first inoculated onto fresh 90 mm PDA plates for activation. Once the mycelium reached approximately 6 cm in diameter, with no visible browning at the center, four mycelial plugs were taken from the colony edges using a 0.5 cm agar punch in a cross-sectional pattern. These activated *I. obliquus* mycelial plugs were then inoculated into 50 ml of liquid PDA medium and cultured in a shaking flask at 28°C with shaking at 160 rpm for 6–7 days, producing the first-stage seed culture.

The 50 ml first-stage seed culture was subsequently transferred into a sterile homogenizer cup and homogenized twice for 15 s each, generating a homogenized first-stage seed culture. This homogenized culture was transferred into 100 ml of fresh liquid PDA medium in a 250 ml Erlenmeyer flask and cultured under the same conditions for an additional 6–7 days. Then, the mycelia were harvested under sterile conditions using gauze and washed three times with sterile deionized water. To remove excess moisture, the mycelia were pressed with sterile forceps, and 18 g of mycelial mass was weighed. Sterile deionized water was then added to adjust the total weight to 100 g, and the mixture was homogenized three times for 15 s each to prepare the second-stage seed culture. This homogenized second-stage culture was used as the subsequent seed culture.

### Effect on the Growth of *I. obliquus*

In the plate experiment, four treatment groups (H&Z group, H group, Z group, and PDA group) were established by supplementing the PDA medium with H&Z inducers, H inducers, Z inducers, and PDA inducers, respectively. Pure PDA medium was used as the control. This experiment primarily aimed to evaluate the effect of MEP2000 fermentation broth, after five rounds of rejuvenation, on the mycelial growth of *I. obliquus*. Using a 0.5 cm agar punch, mycelial plugs were taken from the edges of *I. obliquus* seed colonies and transferred to the center of five different culture plates. The plates were incubated in an inverted position at 28°C in a constant-temperature incubator, with five replicates per treatment. Colony diameter was measured every 72 h, and photographs were taken. The colony growth diameter was determined using the cross-sectional method to calculate the mycelial growth rate, and mycelial vigor was recorded.

In the shake-flask fermentation experiment, 45 ml of freshly prepared PDB medium was supplemented with 5 ml of the corresponding fermentation broth inducer, while 50 ml of pure PDB liquid medium was used as the control. Subsequently, 1 ml of *I. obliquus* secondary seed culture was added to each group, with three replicates per treatment. Samples were collected after 6 and 12 days of shake-flask fermentation. The mycelium was harvested by vacuum filtration, washed three times with distilled water, and dried at 50°C to a constant weight. The dry weight of the mycelium for each group was then measured .

### Effect on the Polysaccharide and Triterpenoid Content of *I. obliquus*

The polysaccharide content was determined using the phenol-sulfuric acid colorimetric method, with glucose as the standard. The *x*-axis represented the glucose concentration, and the *y*-axis represented the absorbance. A microplate reader was used to measure the absorbance at 490 nm, and a glucose standard curve was generated. Exactly 25 mg of dried sample powder from each group in 1.4 was weighed into a 2 ml centrifuge tube, and 1 ml of 1 M NaOH solution was added. The mixture was incubated in a 60°C water bath for 1 h, followed by centrifugation at 10,000 ×*g* for 10 min. A suitable volume of the supernatant was diluted 100-fold, and 100 μl of the diluted solution was transferred to a glass test tube. Then, 0.5 ml of 6% phenol solution was added and gently mixed. Subsequently, 3 ml of concentrated sulfuric acid was slowly added and thoroughly mixed. The mixture was heated in a boiling water bath for 30 min, cooled to room temperature, and the absorbance at 490 nm was measured. The polysaccharide content was calculated using the standard curve equation.

To determine the triterpenoid content, 0.1 g of mycelial powder from each sample was placed into a 100 ml distillation flask, and 10 ml of ethyl acetate was added. Reflux extraction was performed for 2 h, after which the filtrate was collected and concentrated to a final volume of 2 ml. The concentrated solution was then filtered through a 0.22 μm organic phase filter membrane and analyzed by GC-MS . The GC-MS conditions were as follows: Column: Agilent HP-5MS 19091S-433 (30 m × 250 μm × 0.25 μm); temperature program: starting at 150°C, held for 1 min; increased to 280°C at a rate of 30°C/min, then held for 24 min. Helium was used as the carrier gas, with a flow rate of 1 ml/min, and split injection was conducted at a ratio of 10:1, with an injection temperature of 250°C. MS conditions included an electron impact ion source with an ion source temperature of 230°C, an interface temperature of 250°C, electron energy of 70 eV, and a mass scan range of *m/z* 30–500.

### RT-qPCR Analysis of Polysaccharide Synthesis Genes in *I. obliquus*

Mycelial samples of *I. obliquus* were collected after 6 and 12 days of shake-flask fermentation from both the control group and the group treated with the H&Z inducer. The mycelia were washed three times with sterile deionized water, and excess moisture was removed using sterile filter paper. Approximately 200 mg of each sample was used for RNA extraction, which was carried out using the FastPure Cell/Tissue Total RNA Isolation Kit V2 (Vazyme, China) according to the manufacturer’s instructions. After RNA extraction, the RNA concentration was measured using a NanoDrop spectrophotometer (Thermo Fisher Scientific), and RNA integrity was confirmed by 1.5% agarose gel electrophoresis.

Reverse transcription was performed with Vazyme’s HiScript II Q RT SuperMix for qPCR, following the standard protocol provided by the manufacturer. Quantitative PCR (qPCR) was subsequently conducted using the ChamQ SYBR qPCR Master Mix (Vazyme). The qPCR detection program followed the kit's standard procedure, with 18S rRNA serving as the internal reference gene. The primer sequences used for the targeted genes are provided in Table 1.

### Data Analysis

Excel 2021 was utilized to calculate the mean and standard deviation for each treatment. GraphPad Prism 9.5 was used to create grouped bar charts. One-way ANOVA was employed to analyze the effects of different treatments on mycelial growth, polysaccharide content, and other measured parameters.

## Results and Analysis

### Growth of MEP2000 on Different Rejuvenation Media

**Fig. 1:**
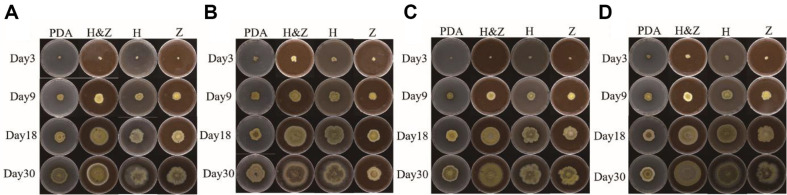
Growth of MEP2000 on various media across domestication generations. (**A**) 2nd-generation domestication. (**B**) 3rd-generation domestication. (**C**) 4th-generation domestication. (**D**) 5th-generation domestication. PDA: MEP3000 was planted on PDA medium as unrejuvenated control. H&Z: MEP3000 was planted on PDA medium with 6 g/l fruiting body powder and 6 g/l birch bark powder. H: MEP3000 was planted on PDA medium with 6 g/l birch bark powder. Z: MEP3000 was planted on PDA medium with 6 g/l fruiting body powder. The plates were all incubated in an inverted position at 28°C in a constant-temperature incubator.

The growth performance of MEP2000 from the second to the fifth generation on various media is shown in Fig. 1. Under identical culture conditions, MEP2000 demonstrated the fastest growth on the H&Z medium, with colonies exhibiting more uniform edges, and this was significantly different from the PDA medium (unrejuvenated control). Growth on the H medium was the second fastest, with colonies expanding at a relatively rapid rate. In contrast, the Z medium displayed a slower growth rate, similar to the unrejuvenated control. Colonies grown on the H&Z medium were particularly regular in shape, forming circular colonies, while colonies on the other media exhibited irregular morphologies. By the fifth generation (Fig. 1D), at 18 days, colonies on the H&Z medium developed dense, aerial mycelium, forming a ring-like raised structure. As cultivation progressed, the aerial mycelium gradually spread outward.

### Effect of MEP2000 Fermentation Broth on the Growth of *I. obliquus*

**Fig. 2:**
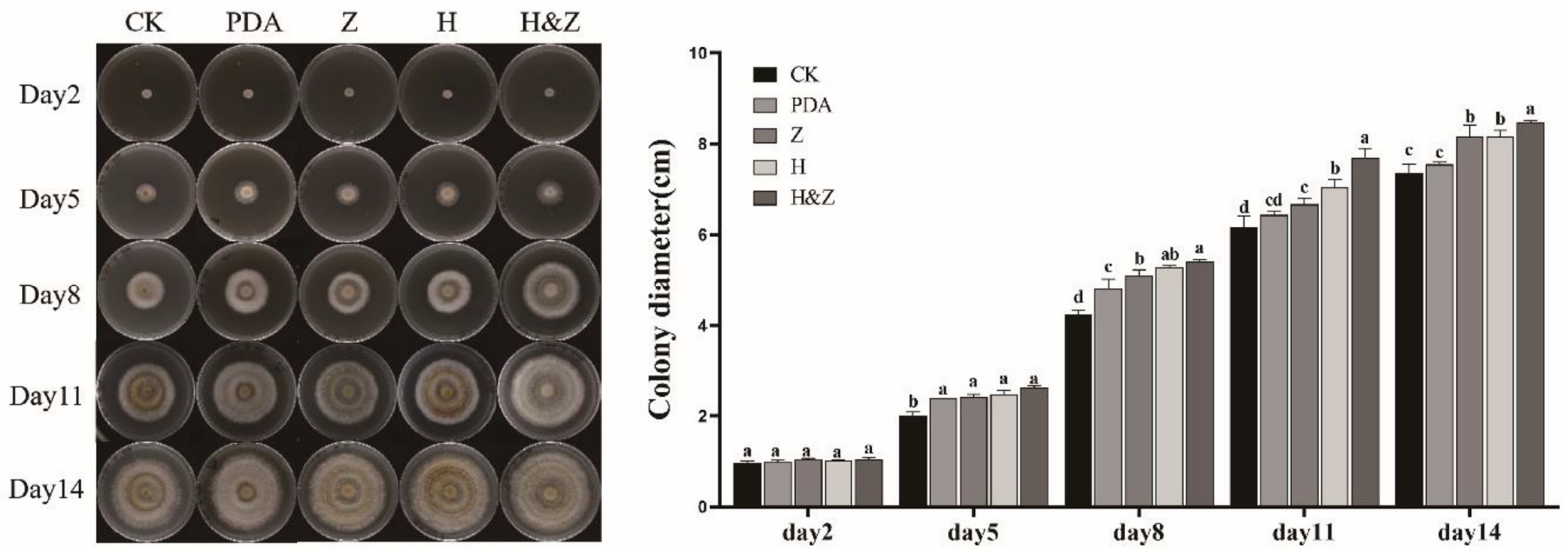
Growth of *I. obliquus* on various media plates. (**A**) Mycelial growth on different media plates. (**B**) Histogram of mycelial growth diameters for each medium. CK: *I. obliquus* was planted on PDA medium without adding MEP2000 fermentation broth. PDA: *I. obliquus* was planted on PDA medium supplemented with PDA inducers (unrejuvenated control). Z: *I. obliquus* was planted on PDA medium supplemented with Z inducers. H: *I. obliquus* was planted on PDA medium supplemented with Z inducers. H&Z: *I. obliquus* was planted on PDA medium supplemented with H&Z inducers. The plates were all incubated in an inverted position at 28°C in a constant-temperature incubator. Different letters indicate significant differences between treatments according to one-way ANOVA (*p* < 0.05).

*I. obliquus* demonstrated growth on all five tested media, though the mycelial growth rates varied across treatments (Fig. 2). On PDA plates supplemented with the H&Z inducer, the mycelial extension of *I. obliquus* was faster, and browning of the mycelium occurred more slowly. The growth rates, ranked from highest to lowest, were as follows: H&Z group > H group > Z group > PDA group (unrejuvenated control) > control group (CK). The H&Z group exhibited the densest mycelial growth and the fastest expansion, showing a significant difference from the control group after 5 days of cultivation. By the 8th day, the H&Z, H, and Z groups showed highly significant differences compared to unrejuvenated control and the control group.

**Fig. 3:**
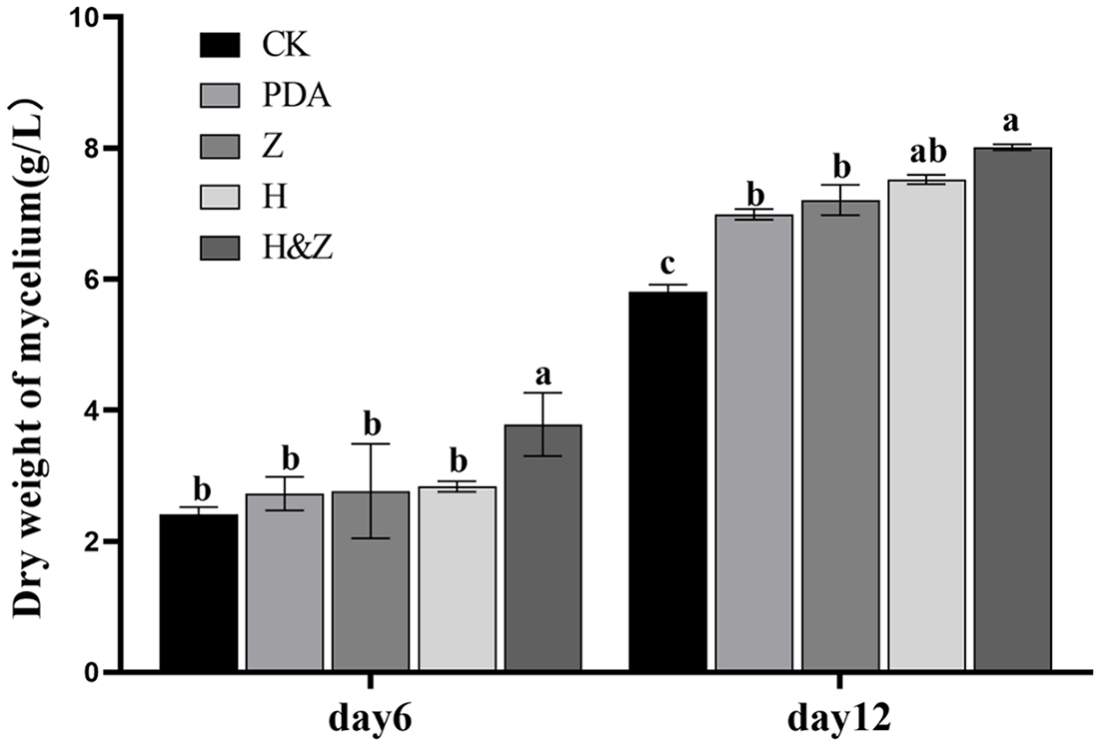
Effect of various fermentation media on the biomass production of *I. obliquus*. CK: *I. obliquus* was planted on PDB medium without adding MEP2000 fermentation broth. PDA: *I. obliquus* was planted on PDB medium supplemented with PDA inducers (unrejuvenated control). Z: *I. obliquus* was planted on PDB medium supplemented with Z inducers. H: *I. obliquus* was planted on PDB medium supplemented with Z inducers. H&Z: *I. obliquus* was planted on PDB medium supplemented with H&Z inducers. They were all cultured in shaking flasks at 28°C with 160 rpm. Different letters indicate significant differences between treatments according to one-way ANOVA (*p* < 0.05).

Under identical culture conditions, significant differences in biomass were observed across the fermentation broth groups with different additives. After 6 and 12 days of fermentation, the H&Z group consistently yielded the highest biomass (Fig. 3). After 6 days, the mycelial biomass in the control group was 0.12 g, while the H&Z group reached 0.19 g, which was 1.58 times higher than the control. The H group produced 0.14 g, 1.17 times the control, and the Z group yielded 0.138 g, 1.15 times the control. The mycelial biomass in the PDA group was 0.136 g, while the mycelial biomass in the H&Z, H, and Z groups was 1.4, 1.04, and 1.01 times that of the PDA group, respectively. A significant difference was observed between the H&Z group and the control or PDA. After 12 days of fermentation, the control group achieved a biomass of 0.29 g, while the H&Z group reached 0.40 g, 1.38 times the control. The H group produced 0.376 g, 1.3 times the control, and the Z group yielded 0.360 g, 1.24 times the control. The mycelial biomass in the PDA group was 0.35 g, while the mycelial biomass in the H&Z group was 1.14 times that of the unrejuvenated control. There were significant differences between the H&Z group and the control group, as well as the unrejuvenated control.

### Effect of MEP2000 Fermentation Broth on the Accumulation of Extracellular Polysaccharides in *I. obliquus*

The standard curve equation for polysaccharides was y = 177.82x 17.942, with an R² value of 0.996. Polysaccharide content was expressed as the mass of glucose per gram of dry biomass. After 6 days of liquid shake-flask fermentation, the H&Z group exhibited the highest polysaccharide content in the mycelium, reaching 205.08 mg of polysaccharides per gram of mycelium. The H group ranked second with 141.85 mg, followed by the Z group with 120.67 mg, the control group with 114.75 mg, and the PDA group with 100.12 mg. Compared to the control, the H&Z, H, and Z groups had polysaccharide levels 1.79, 1.24, and 1.05 times higher, respectively. The H&Z, H, and Z group were 2.05, 1.42, and 1.21 times higher than the PDA group (the unrejuvenated control), respectively.

**Fig. 4:**
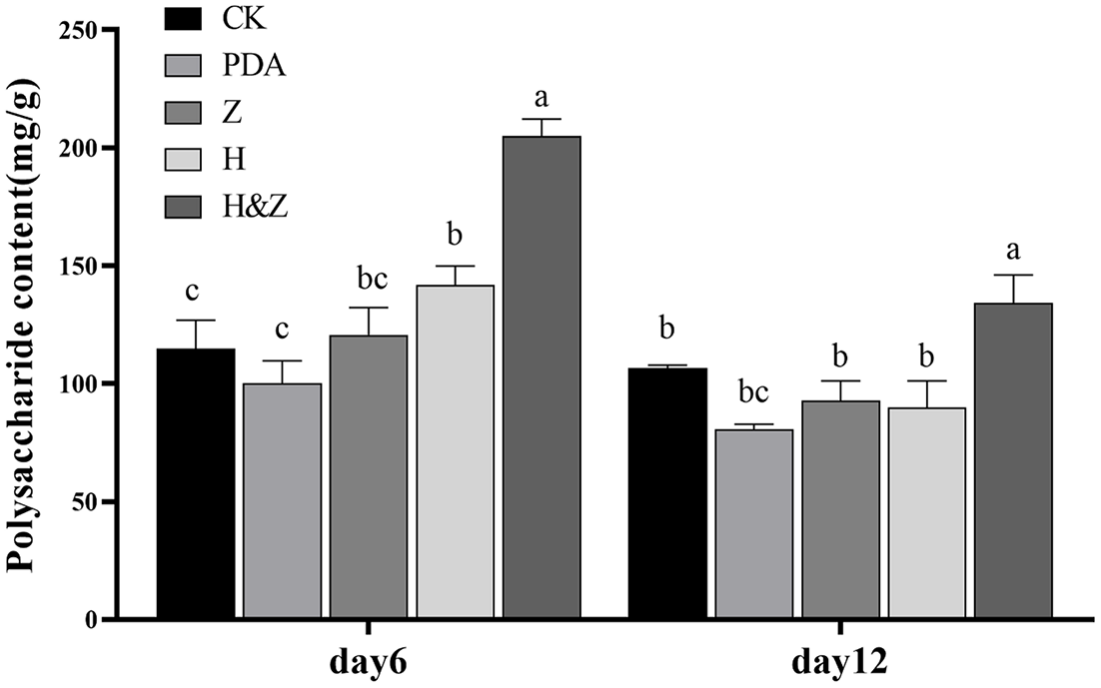
Effect of various fermentation media on the polysaccharide content of *I. obliquus*. CK, PDA, Z, H, and H&Z were as shown in Fig. 3. Different letters indicate significant differences between treatments according to one-way ANOVA (*p* < 0.05).

After 12 days of fermentation, the H&Z group continued to exhibit the highest polysaccharide content, measuring 134.19 mg per gram of mycelium, followed by 90.01 mg in the H group, 92.87 mg in the Z group and 80.68 mg in PDA group. These values were 1.25, 0.84, 0.87 and 0.75 times that of the control, respectively and the H&Z, H, and Z were 1.66, 1.12, and 1.15 times higher than unrejuvenated control, respectively. Overall, the polysaccharide content in the mycelium of *I. obliquus* was higher on day 6 compared to day 12 (Fig. 4).

### Effect of MEP2000 Fermentation Broth on the Triterpenoid Content in *I. obliquus*

GC-MS analysis of the ethyl acetate extracts from the mycelia after 12 days of fermentation detected a total of 19 compounds, including squalene, ergosterol, lanosterol, and inotodiol, all of which are triterpenoid compounds (Table 2). Lanosterol was used as the standard for quantitative analysis, with its concentration directly proportional to the peak area. A comparison of peak areas across the samples revealed that the content of all 19 compounds increased in the H&Z group, with the inotodiol content reaching 3.88 times that of the control. In the PDA, H, and Z groups, 12, 18, and 17 compounds, respectively, showed significant increases, with inotodiol levels 2.27, 2.42, and 2.28 times higher than the control. These findings indicate that the H&Z group had the most pronounced effect on enhancing the content of bioactive triterpenoid compounds in *I. obliquus*.

### Effect of MEP2000 Fermentation Broth on the Expression of Polysaccharide Synthase Genes in *I. obliquus*

On the 6th and 12th days of fermentation, the H&Z group exhibited the highest polysaccharide content in *I. obliquus*. To explore the role of the H&Z inducer in the regulation of polysaccharide biosynthesis, we conducted RT-qPCR to quantify the expression of genes associated with polysaccharide synthesis . Samples from the control and H&Z groups were collected on the 6th and 12th days, and total RNA was extracted and reverse transcribed into cDNA. RT-qPCR was then performed to analyze the expression of polysaccharide synthase genes.

**Fig. 5:**
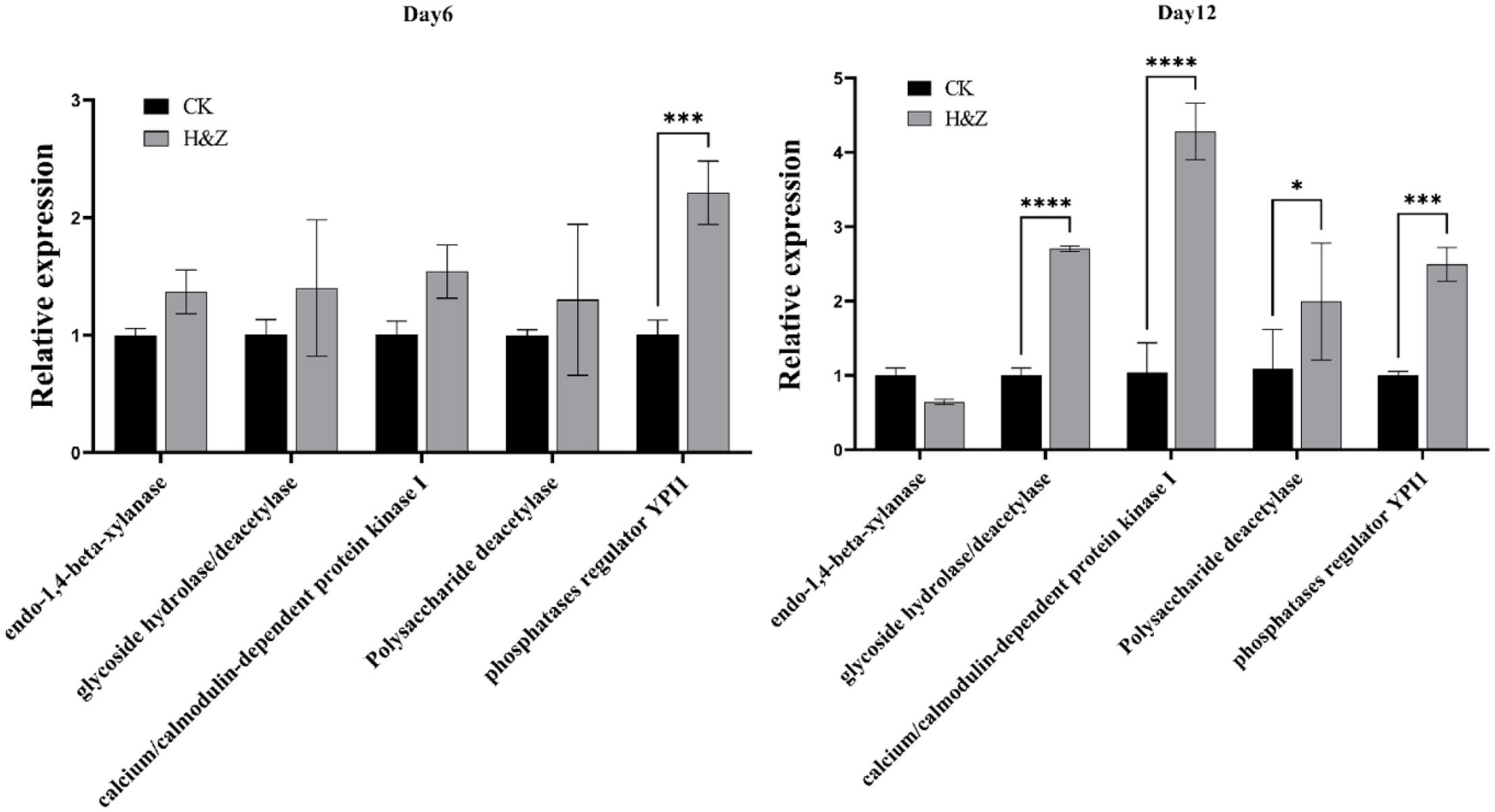
Effect of MEP2000 fermentation broth on the gene expression of polysaccharide synthase in *I. obliquus* after 6 and 12 days of shake-flask fermentation. CK: *I. obliquus* was planted on PDB medium without adding MEP2000 fermentation broth. H&Z: *I. obliquus* was planted on PDB medium supplemented with H&Z inducers. Different letters indicate significant differences between treatments according to one-way ANOVA (*p* < 0.05).

The RT-qPCR results, as shown in Fig. 5, indicated that on day 6 of fermentation, the relative expression of polysaccharide biosynthesis-related genes was elevated in the H&Z group, with a significant increase observed in phosphatases regulator YPI1. By day 12, the expression levels of most genes had further increased, with significant upregulation observed in glycoside hydrolase/deacetylase, calcium/calmodulin-dependent protein kinase I, glycoside hydrolase/deacetylase, and phosphatases regulator YPI1.

## Discussion

Strain degeneration often manifests through changes in growth characteristics and alterations in metabolic product profiles . During the rejuvenation process, we found that MEP2000 exhibited the fastest growth and the most consistent colony morphology when cultured on the H&Z medium, indicating that this medium is optimal for MEP2000 growth. Our experimental results showed that the rejuvenated MEP2000 fermentation broth restored its growth-promoting ability and enhanced polysaccharide biosynthesis in *I. obliquus*. Among the tested media, the MEP2000 fermentation broth cultured on the H&Z medium for five rounds resulted in the fastest mycelial growth in *I. obliquus*, followed by the H medium, with the Z medium also showing a positive effect. This effect may be attributed to the dependence of *I. obliquus* fruiting bodies on birch tree-derived nutrients for growth; while MEP2000 is an endophytic fungus of *I. obliquus*, it might utilize components from both the birch tree and *I. obliquus* itself to sustain metabolite production, thereby significantly promoting growth and polysaccharide accumulation in *I. obliquus*.

In our study, we also observed that during the liquid fermentation of *I. obliquus*, the polysaccharide content was higher on day 6 compared to day 12. This reduction over time is likely due to nutrient depletion, forcing the fungus to metabolize its own polysaccharides for continued growth. This pattern of intracellular polysaccharide accumulation is consistent with the findings of Zhang *et al*., who reported that extracellular polysaccharide levels in *I. obliquus* increased during the early stages of fermentation, peaking on day 7, and then gradually declined. However, in our study the MEP2000 fermentation broth cultured on the H&Z medium weakened the dependence of *I. obliquus* on polysaccharides as nutrition during the later stages of fermentation.

At the molecular level, the accumulation of polysaccharides is reflected in the differential expression of polysaccharide synthase genes. Transcriptomic analysis of *I. obliquus* treated with MEP2000 fermentation broth revealed the upregulation of several polysaccharide synthesis-related genes . We selected five of these upregulated genes for RT-qPCR analysis and confirmed that *I. obliquus* treated with H&Z fermentation broth showed increased expression of polysaccharide synthase genes, supporting the hypothesis that the H&Z inducer enhances polysaccharide synthesis. By day 12, significant upregulation of glycoside hydrolase/deacetylase, calcium/calmodulin-dependent protein kinase I, glycoside hydrolase/deacetylase, and phosphatases regulator YPI1 was observed, suggesting that while overall polysaccharide synthesis declined by day 12, the inducer regulated gene expression to maintain polysaccharide accumulation.

Our GC-MS analysis identified 19 compounds, and a comparison of peak areas revealed that most compounds in the samples treated with fermentation broth inducers were present at higher concentrations than in the control group (Table 2). The degree of compound increase varied depending on the type of inducer used. The H&Z group exhibited the greatest increase, both in the number of compounds and their concentrations. Triterpenoids are critical bioactive compounds in *I. obliquus* , with squalene acting as a precursor in triterpenoid synthesis, and lanosterol providing the structural scaffold for active triterpenoids. Inotodiol, a lanostane-type triterpenoid, is recognized as the primary anticancer compound in *I. obliquus* . The elevated levels of these compounds suggest that the H&Z fermentation broth effectively promotes the biosynthesis of bioactive triterpenoids in *I. obliquus*. Many studies have shown that endophytic fungi can produce a variety of secondary bioactive metabolites that provide host plants tolerance to various biotic and abiotic stresses, promote nutrient acquisition, enhance their defenses against pathogens and pests, and modulate the synthesis of secondary metabolites [38, 39]. In our experiment, MEP3000 grew on different revitalization medium and may form different secondary metabolites, which had different effects on the accumulation of triterpenoids in *I. obliquus*. Therefore, the promotion of the synthesis of triterpenes in H, Z, and H&Z groups will be inconsistent.

This study highlights that MEP2000 differentially utilizes supplemented media components, with the combination of *I. obliquus* fruiting body powder and birch bark powder proving to be the most effective for its rejuvenation. After being revitalized on this medium, the fermentation broth inducers exhibited the most pronounced effects on promoting both the growth of *I. obliquus* and the synthesis of polysaccharides and triterpenoids. These findings offer a valuable foundation for the future application of high-activity *Acremonium* strains in the large-scale production of *I. obliquus*.

## Figures and Tables

**Table 1:** Primer information used for RT-qPCR analysis.

Gene name	Primer sequence (5’→3’)
Endo-1,4-beta-xylanase	F: CTACTACGCCCATCGTATCATC R: GATTTCGGCATCCACGAATAAG
Glycoside hydrolase/deacetylase	F: TGTGCCTTCCTCATCTGTAAAC R: GTTATCGACGCCAAGGGAATAG
Calcium/calmodulin-dependent protein kinase I	F: AGAGACGTTGGATGGGACTA R: GTATTTCTTTGCCCGGGTTTG
Glycoside hydrolase/deacetylase	F: CACGATTCGCAGGCATACTA R: CAGTCTCATTACCTCGCTGAAG
Phosphatases regulator YPI1	F: GAGCTTTCGGATGACGAGTATG R: GCCAGGATTGTCGCTCTTT
18S rRNA	F: CCTTGACACTACGAGGGATAAC R: TCCATCATCAATCCCGAACAA

**Table 2:** Changes in peak areas of terpenoids induced by various additives.

Compound No.	tR (min)	Compound name	Relative content of terpenoids in *I. obliquus* (×10^3^)				
			CK	PDA	H	Z	H&Z
1	10.286	9-Hexadecenoic acid	121.48±5.233	135.48±11.212	186.67±18.081	228.38±17.065	181.94±16.293
1	10.286	9-Hexadecenoic acid	121.48±5.233^c^	135.48±11.212^c^	186.67±18.081^b^	228.38±17.064^a^	181.95±16.294^b^
2	10.673	Squalene	157.09±8.247^c^	204.69±10.620^b^	332.77±19.922^a^	344.28±20.131^a^	308.98±20.742^a^
3	11.825	1-Heptatriacotanol	59.61±1.648^c^	73.845±4.187^b^	88.39±5.223^a^	79.368.1±6.09^ab^	83.822±5.19^ab^
4	12.087	Cholesteryl myristate	51.106±1.530^c^	70.924±4.549^b^	71.90±3.667^b^	87.53±7.037^a^	64.39±3.969^b^
5	12.474	Ergosta-5,7,9(11),22-tetraen-3-ol	358.09±10.919^c^	343.21±11.950^c^	510.09±19.877^b^	608.89±26.145^a^	584.89±26.457^a^
6	12.809	Anthiaergostan-5,7,9,16,22-penten	136.68±6.798^c^	130.45±7.911^c^	217.82±14.522^b^	243.55±13.28^ab^	273.35±19.419^a^
7	15.29	Dehydroergosterol,O-TMS	476.69±19.513^c^	471.96±24.837^c^	684.79±33.565^b^	766.32±43.67^ab^	817.36±43.398^a^
8	15.981	Ergosta-5,8,22-trien-3-ol, (3β,22E)-	2504.42±140.229^b^	2178.36±126.31^bc^	3141.38±193.22^a^	1829.10±110.49^c^	3312.09±188.29^a^
9	16.243	Ergosta-5,7,25(27)-trienol	66.84±3.872^d^	100.25±3.832^c^	221.23±22.314^a^	155.01±11.441^b^	153.31±4.214^b^
10	17.353	Stigmasta-7,24(28)-dien-3-ol, (3β,5α)-	124.23±6.828^c^	91.42±5.855^d^	136.99±7.893^bc^	151.85±9.570^b^	227.51±6.597^a^
11	17.468	γ-Ergostenol	156.64±8.682^b^	115.69±7.644^c^	155.11±7.052^b^	183.84±10.382^a^	195.91±14.088^a^
12	18.012	9-cis-retinal	50.27±3.301^c^	63.21±1.908^b^	62.58±2.772^b^	53.60±3.686^c^	79.24±3.838^a^
13	18.274	11α-hydroxyprogesterone	52.90±3.531^c^	87.22±5.141^b^	125.143±8.130^a^	134.29±9.329^a^	137.15±9.036^a^
14	18.745	Lanosterol	1843.02±102.77^c^	2551.60±145.119^b^	3017.78±169.15^a^	3028.11±170.73^a^	3412.63±186.95^a^
15	20.18	Lanost-8-ene-3,7-dione, (13α,14β,17α)-	40.83±1.508^d^	67.75±4.369^b^	47.70±2.274^cd^	51.30±3.187^c^	85.11±2.863^a^
16	20.64	1,4-dichloro-2-fluorobenzene	50.21±1.913^d^	62.67±4.966^c^	65.46±6.499^c^	103.14±2.649^a^	81.48±4.934^b^
17	25.467	Eburicol	3149.63±139.152^d^	6366.54±399.337^c^	8150.31±542.9^b^	7177.35±438.8^bc^	10798.55±722.081^a^
18	26.294	Inotodiol	995.55±52.612^c^	2256.84±157.443^b^	2412.36±176.15^b^	2271.09±132.43^b^	3859.08±267.391^a^
19	28.074	Tris(2,4-di-tert-butylphenyl) phosphate	1480.29±69.524^c^	1280.42±70.482^c^	1833.34±100.20^b^	2927.36±159.41^a^	2637.07±130.486^a^

Different letters indicate significant differences between treatments according to one-way ANOVA (*p* < 0.05).
